# Rupture Predictions of Notched Ti-6Al-4V Using Local Approaches

**DOI:** 10.3390/ma11050663

**Published:** 2018-04-25

**Authors:** Mirco Peron, Jan Torgersen, Filippo Berto

**Affiliations:** Department of Industrial and Mechanical Engineering, Norwegian University of Science and Technology, Richard Birkelands vei 2b, 7491 Trondheim, Norway; jan.torgersen@ntnu.no (J.T.); filippo.berto@ntnu.no (F.B.)

**Keywords:** notched Ti-6Al-4V, strain energy density, theory of critical distances, SED, TCD, tensile strength, tensile strength prediction

## Abstract

Ti-6Al-4V has been extensively used in structural applications in various engineering fields, from naval to automotive and from aerospace to biomedical. Structural applications are characterized by geometrical discontinuities such as notches, which are widely known to harmfully affect their tensile strength. In recent years, many attempts have been done to define solid criteria with which to reliably predict the tensile strength of materials. Among these criteria, two local approaches are worth mentioning due to the accuracy of their predictions, i.e., the strain energy density (SED) approach and the theory of critical distance (TCD) method. In this manuscript, the robustness of these two methods in predicting the tensile behavior of notched Ti-6Al-4V specimens has been compared. To this aim, two very dissimilar notch geometries have been tested, i.e., semi-circular and blunt V-notch with a notch root radius equal to 1 mm, and the experimental results have been compared with those predicted by the two models. The experimental values have been estimated with low discrepancies by either the SED approach and the TCD method, but the former results in better predictions. The deviations for the SED are in fact lower than 1.3%, while the TCD provides predictions with errors almost up to 8.5%. Finally, the weaknesses and the strengths of the two models have been reported.

## 1. Introduction

The adoption of Ti-6Al-4V alloy is widespread in advanced engineering fields, such as military, aerospace, automotive, and naval applications, due to its very good static and fatigue properties, high strength-to-mass ratio, and excellent wear resistance, including at high temperatures [1,2]. Moreover, Ti readily forms a titanium dioxide (TiO_2_) outer layer that assures its passivity in an oxidizing environment, thus determining a high corrosion resistance and guaranteeing a great reliability of Ti-6Al-4V in applications in which corrosion is one of the main issues, such as biomedical devices [3]. In all these applications, geometrical discontinuities (notches) negatively affect the fracture and fatigue strength [4,5,6,7]. In recent years, due to its fundamental importance, the tensile strength assessment of notched components has been widely investigated in order to define an efficient criterion to perform a reliable static assessment. Many researchers have attempted to predict static strength of components weakened by different notches leveraging on linear elastic fracture mechanics (LEFM) theory, and in particular on notch stress intensity factors (NSIFs). The method has revealed to be accurate in predicting the tensile strength of components weakened by sharp notches and by cracks, either under uniaxial or mixed mode loading [8,9,10,11,12,13,14]. In addition, when components are weakened by rounded notches, Lazzarin and Filippi have proposed a generalized stress intensity factor as a failure governing factor when the effect of stress redistribution due to the notch root radius cannot be neglected [15]. However, the development of the aforementioned methods in assessing real components has been limited by the need to accurately determine the stress distribution ahead of the notches to correctly perform the static assessment, leading to time-consuming stress field analyses. In addition, the main drawback lies with the geometry dependence of the NSIF-based criteria (their units are MPa(m)^β^). The exponent *β* depends on the notch opening angle, according to the expression *β* = 1 − *λ*_1_, in which *λ*_1_ is the Williams’ eigenvalue [16]. These methods are thus difficult to use, because ad-hoc material properties have always to be determined experimentally.

To overcome these limitations, Lazzarin and Zambardi [17] formalized the strain energy density (SED) approach, in which the tensile strength of notched component is stated to be reached when the strain energy, $\bar{W}$, averaged over a finite volume centered at the stress raiser, reaches its critical value *W_c_*. Both the critical SED value and the radius *R_c_* of such a volume have been found to depend only on material properties [4]. From the introduction of the Absorbed Specific Fracture Energy (ASFE) for the fracture assessment of low and medium strength structural materials [18], several energy-based approaches have in fact been developed. For example, Sih et al. [19,20,21] proposed a point-related criterion, stating the failure to be controlled using a critical value of the strain energy density factor *S*. However, the volume-based approach proposed by Lazzarin and Zambardi represents a great development of all the previous strain energy density-based approaches. This method has been revealed to successfully predict both the tensile and fatigue strength of different notched materials subjected to both uniaxial and mixed-mode loading [22,23,24,25,26,27,28,29,30,31]. Piccotin et al., for example, assessed with good agreements the tensile strength under mode I, II, and mixed mode I + II of polyurethane foams characterized by different densities and weakened by several notch geometries [32]. It is worth also noting that the SED approach represents an interesting development of the so-called theory of critical distances (TCD). The TCD is in essence a set of methodologies, all of which use a material length parameter (the critical distance, *L*) when performing fracture or fatigue assessments of notched components. It owes its name to Taylor [33,34], but the origin of the TCD is found in the works of Neuber [35] and Peterson [36]. According to this approach, failure occurs when the stress averaged over a line (line method, LM) or calculated at a certain distance from the notch root (point method, PM) equals the characteristic strength of the material *σ*_0_. These TCD methodologies have been successfully applied both to fatigue and fracture assessment of different materials, resulting in a good agreement between the experimental and predicted strength values [37,38,39]. This paper deals with contributing to the development of the analysis of notched components, and is very important for defining which methods can be used to predict their failure. To this aim, a comparison between the tensile strength predictions provided by two of the most affirmed criteria available in literature has been carried out. Within this scope, the tensile strength of wrought Ti-6Al-4V specimens weakened by semi-circular and blunt V-notch with a notch root radius equal to 1 mm have been estimated by means of the SED approach and the TCD criterion. Moreover, un-notched specimens have been tested to determine the mechanical properties required by the two models. Although both the approaches provide satisfactory estimations, the SED predictions are characterized by lower discrepancies. In addition, the strengths and weaknesses of the two approaches are reported.

## 2. Materials and Methods

**Figure 1:**
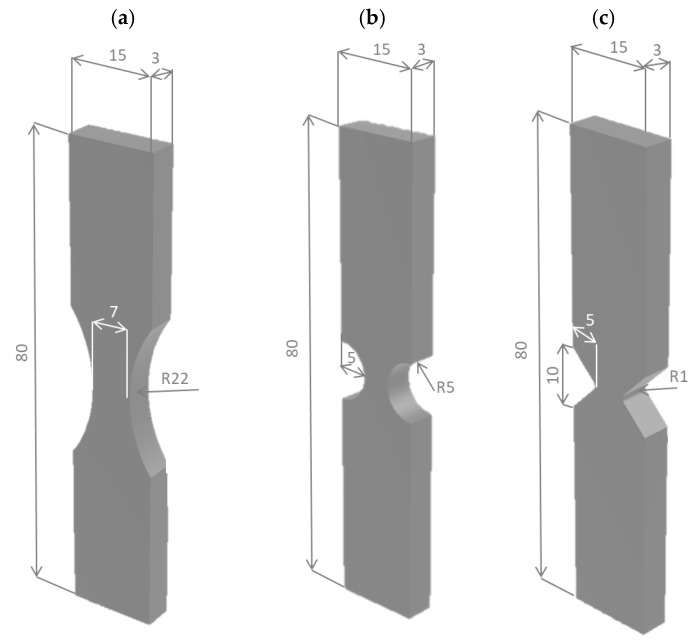
Schematic representation of the specimen geometries.

To fabricate the specimens, a grade 5 titanium alloy (Ti-6Al-4V) sheet of 3 mm thick was first provided by CRP MECCANICA S.r.l. (Modena, Italy). Then, the drawings of un-notched and notched specimens were given to a high-precision 2D CNC laser cutting machine. The dog-bone un-notched specimens (Figure 1a) were characterized by a net section of 3 mm in thickness and 7 in width and by a connecting radius between the net and gross sections large enough to avoid any effect of stress concentration (*ρ* = 22 mm). In addition, with the aim of assessing the reliability of two of the most robust criteria available in literature about the prediction of the tensile strength of components weakened by different notch geometries, two different notched components have been tested. Samples have been manufactured with very dissimilar notch geometries, i.e., a semi-circular notch and a blunt V-notch (Figure 1b,c, respectively). The former was characterized by a connecting radius of 5 mm, whereas the latter was characterized by a V-notch depth of 5 mm, an opening angle equal to 90°, and a notch root radius *ρ* = 1 mm. The dimension of the notch-tip radius was confirmed by several measurements obtained by means of an optical microscope and dedicate software.

Tensile tests (ten per each specimen geometries) were carried out by using a universal MTS machine (50 kN) (MTS, MN, USA), and a low crosshead rate of 0.2 mm/min was used. In particular, dog-bone un-notched specimens have been tested to determine the tensile properties of the material being analysed. The main material parameters, i.e., Young’s modulus *E*, Poisson’s ratio *ν*, 0.2% proof strength *σ*_0.2,_ and ultimate tensile strength *σ_UTS_* have been calculated by means of a MTS 632-85 biaxial extensometer. Their mean values are gathered in Table 1, in which bold numbers represent the average values and numbers in brackets the relative standard deviation, respectively.

## 3. Theoretical Background

### 3.1. Strain Energy Density Criterion

According to the SED approach, the failure of a component is governed by a local parameter such as the total strain energy density; when its value, averaged over a circular control volume of critical radius *R_c_* ahead of a crack or a notch tip, reaches the critical value *W_c_*, the failure occurs [17]. Berto and Lazzarin [4] reported these critical parameters to be only material-dependent. This approach has been extensively used in the assessment of the tensile and fatigue behavior of different materials weakened by several notch geometries [31,40,41], and the results reported excellent prediction capabilities of this approach. From the theory in Ref. [17], critical parameters can be analytically obtained with only few material properties: the ultimate tensile strength of the un-notched material *σ_UTS_*, the fracture toughness *K_IC_*, and the Young’s modulus *E*. In fact, considering Beltrami’s work [42], the following expression can be used to determine the critical value of the total strain energy:

(1) $$W_{c}=\frac{\sigma_{UTS}^{2}}{2E}$$

**Figure 2:**
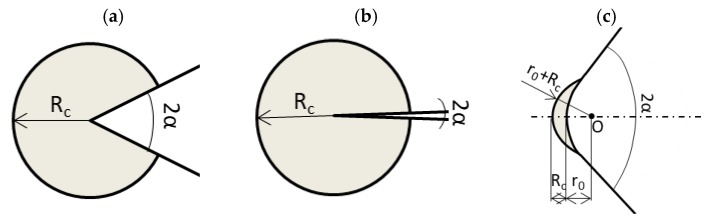
Control volume for sharp V-notch (**a**), crack case (**b**), and blunt V-notch (**c**) under opening mode I loading.

Considering plane problems, the control volume becomes a circular sector or a circle, for V-notches or cracks, respectively (Figure 2a,b), and the critical radius *R_c_* can be expressed as follows [17]:

(2) $$R_{c}=\frac{\left(1+v\right)\left(5-8v\right)}{4\pi }{\left(\frac{K_{Ic}}{\sigma_{UTS}}\right)}^{2}$$

**Figure 3:**
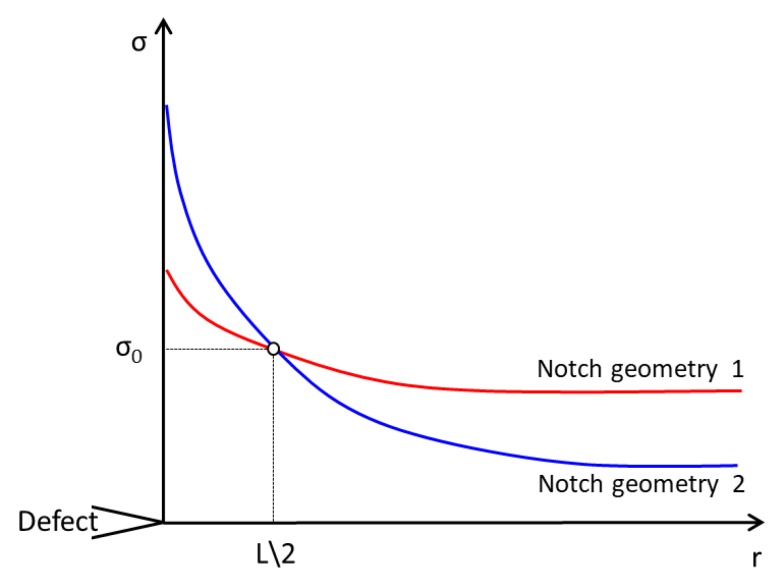
Determination of inherent strength *σ*_0_ and critical distance L leveraging on the so called point method. “Notch geometry 1” and “notch geometry 2” are here used to generally describe two different notch geometries, in which “notch geometry 2” has a more severe notch than “notch geometry 1”.

For a U-notch or a blunt V-notch (Figure 3c), the volume is assumed to be of a crescent shape, in which *R_c_* is the depth measured along the bisector line. The outer radius of the crescent shape is equal to *R_c_* + *r*_0_, in which r_0_ is the distance between the notch tip and the origin of the local coordinate system (Figure 2c). Such a distance depends on the notch-opening angle 2*α*, according to the expression:

(3) $$r_{0}=\rho \frac{\left(\pi -2\alpha \right)}{\left(2\pi -2\alpha \right)}$$

However, in certain circumstances, the analytical formulations provided in Equations (1) and (2) cannot be used for the determination of the critical value of the SED and that of the radius, being all the material parameters required to apply SED approach not always available. The introduction of the FE codes allows one to overcome this shortcoming thanks to their capability to easily determine the strain energy density within a certain control volume. Peron et al. [7], analyzing the tensile strength of PEEK under different environmental conditions, overcame the lack of knowledge of the fracture toughness value determining the critical radius varying the control volume of notched specimens with two different notch radii in a FE code (Ansys^®^) and iteratively computed the SED value until a satisfying convergence was reached. However, with wrought Ti-6Al-4V having been widely studied, in the following the authors has decided to determine the critical radius value using previous studies reporting the fracture toughness, with negligible difference with the critical radius obtained using the procedure described in Ref. [7].

### 3.2. Theory of Critical Distances

The TCD is in essence a set of methodologies, all of which recognize failure in the presence of stress concentration features to be governed not only by the stress and strain at the notch surface but rather by the stress field in the vicinity of the notch. In fact, fracture processes involving crack initiation and propagation are strongly influenced by the absolute volume of material that is experiencing high stresses. In particular, four different methodologies have been developed; two are stress-based, while the other two are based on stress intensity factor. In the following, for sake of simplicity, only the former will be described, whereas for a deeper insight into the latter, the reader should refer to Ref. [34]. The two stress-based methodologies are known as point method (PM) and line method (LM), and the failure is stated to occur whether the stress at a distance *r_c,PM_* from the notch (PM) or averaged over a length *r_c_,_LM_* reaching out from the stress raiser (LM) reaches the so called inherent strength *σ*_0_, i.e.,

(4) $$\frac{1}{r_{c,LM}}\int_{0}^{r_{c,LM}}\sigma (r)\cdot dr=\sigma_{0}$$

Both the distances *r_c,PM_* and *r_c,LM_* are related to the so called critical distance *L*, being *r_c,PM_* and *r_c,LM_* equal to $\frac{L}{2}$ and 2*L*, respectively. Labeled as critical distance by Taylor, *L* is defined as

(5) $$L=\frac{1}{\pi }{\left(\frac{K_{IC}}{\sigma_{0}}\right)}^{2}$$

in which *K_IC_* is the material fracture toughness. Concerning instead the inherent strength, in brittle materials such as ceramics [38], *σ*_0_ is equal to the ultimate tensile stress, *σ_UTS_*, whereas when static failures are preceded by a certain amount of plasticity, σ_0_ is higher than the plain material strength, being $\sigma_{0}=T\cdot \sigma_{UTS}$. It has been found that T is in the range 1.4–3 for polymers [43], and it is typically greater than 3 for metals [44]. A precise interpretation of the meaning of the *T* parameter is still unclear, but it may be related to the material behavior including plasticity and porosity, and thus the inherent strength *σ*_0_ can be determined only by carrying out ad-hoc experiments. Tensile tests need to be carried out on notched specimens weakened by two different notch geometries, and then the stress fields ahead of the notch at the condition of incipient failure need to be assessed, for example, when using finite element codes. Leveraging on the point method, the TCD fundamental parameters *L* and *σ*_0_ can be evaluated as follows: reporting the stress fields as in Figure 3, the inherent strength *σ*_0_ could be defined as the ordinate value at which the two stress distributions intersect; in addition, the abscissa value of the intersection is equal to $\frac{L}{2}$, i.e., *r_c,PM_*.

In the following, once the TCD critical parameters by means of the point method are determined, the tensile strength of the notched specimens will be predicted leveraging on the line method.

## 4. Results

### 4.1. Tensile Tests

The tensile strength for each of the notched geometries is summarized in Table 2, in which bold numbers represent the average values and numbers in brackets the relative standard deviations, respectively. A statistical data analysis has been carried out, and the Chauvenet’s criterion has been applied to delete any potential spurious data.

The results obtained from the tensile tests have then been analysed both in terms of SED and TCD (line method) to assess the reliability of the methods as an engineering tool for predicting the tensile behaviour of notched Ti-6Al-4V. In addition, a comparison between the two criteria have been carried out.

### 4.2. Critical SED Parameters and Synthesis of Tensile Data in Terms of SED

**Figure 4:**
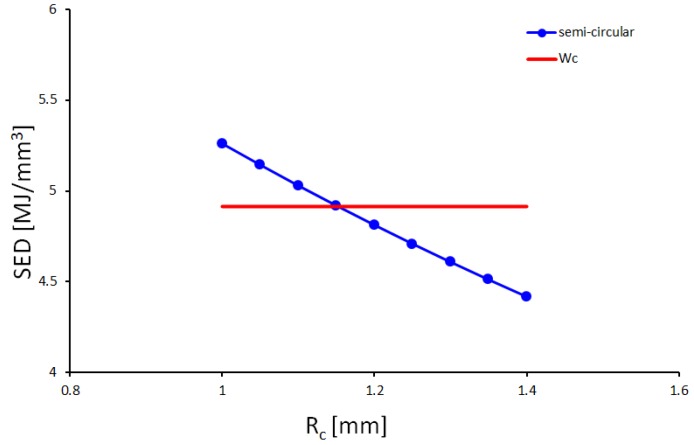
Determination of the critical radius value as the value at which a correspondence between the computed SED value (blue) and that obtained by means of Equation (1) (red) is found.

The determination of the critical value of the strain energy density *W_c_* and that of the radius *R_c_* of the control volume is required for the application of the SED criterion. Concerning the former critical parameter, Equation (1) has been applied in this work and, leveraging on the tensile data obtained from the dog-bone specimens (Table 1), it leads to a *W_c_* value of 4.91 MJ/m^3^. Regarding the critical value of the radius, no fracture toughness tests have been carried out by the authors. Thus, the critical value of the radius has been assessed using the fracture toughness value of 74.2 MPa√m reported in Ref. [45] as input in Equation (2), leading to a critical radius value *R_c_* of 1.19 mm. The value just estimated is in very good agreement with the value that is more accurately obtained according to the procedure briefly mentioned in Section 3.1 and better described in Refs. [7,29]. If the fracture toughness is not available, it is possible to estimate the critical radius using finite element analyses. In this way, the strain energy of specimens weakened by a certain notch geometry can be averaged over control volumes of different critical radii *R_c_*. The critical radius can be estimated as the value at which the computed SED value equals that obtained using Equation (1). In this work, the critical radius has been varied in Ansys^®^ code from 1 to 1.8 mm with a range of 0.05 mm for the semi-circular notched specimen. Comparing the computed SED values (blue line in Figure 4) with that measured using Equation (1) (red line in Figure 4), a critical radius *R_c_* of 1.15 mm has been obtained.

**Figure 5:**
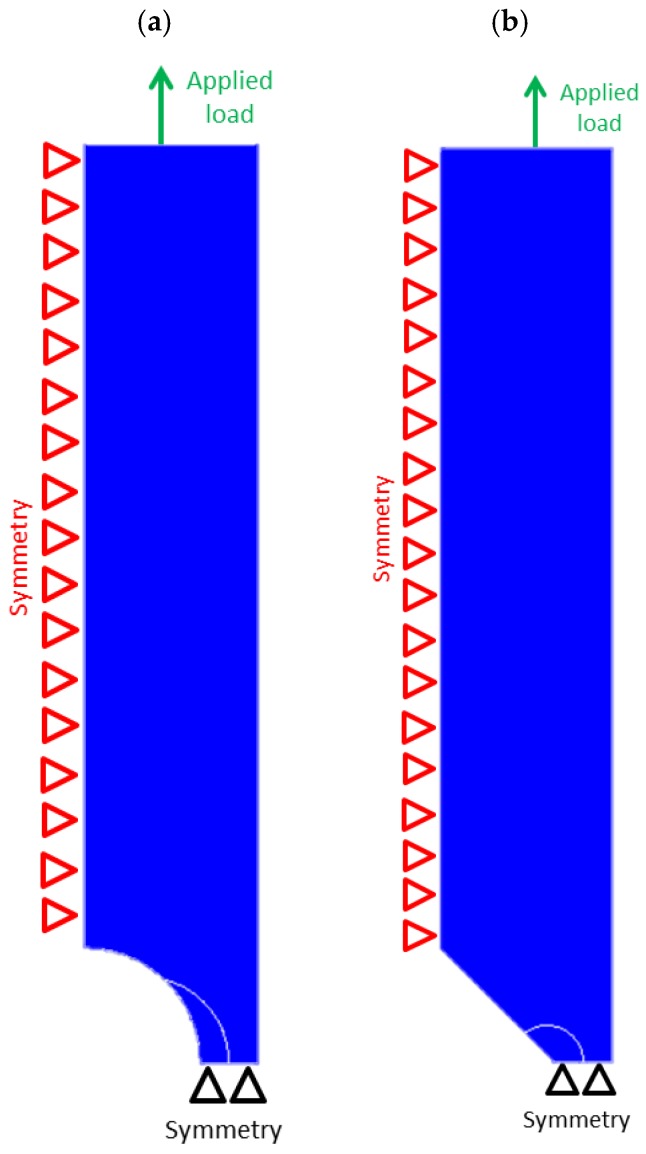
Boundary conditions in FE models for specimens weakened by a semi-circular notch (**a**) and a blunt V-notch (**b**).

Tensile strength predictions of semi-circular and blunt V-notched specimens have been carried out by means of FE modelling. Linear elastic 2D analyses have been carried out using Ansys^®^ code and 8-nodes iso-parametric element plane 183 with plane strain key-option that has been selected. Because the SED approach is mesh insensitive [28,40,46], a coarse mesh has been adopted at the notch tip. In modeling the geometry, the double symmetry was used; thus, only one quarter of the specimens and symmetric boundary conditions were used for vertical and horizontal symmetry lines of the models (Figure 5). The top side of the model was instead able to move along the loading axis.

The failure load has been evaluated as the load that should have been applied in the FE analyses for obtaining a SED value in the control volume (*R_c_* = 1.19 mm) equal to that critical. As the strain energy density is proportional to the square of the applied stress, the predicted tensile strength of semi-circular and blunt V-notch specimens, $\sigma_{UTS,predicted}$, has been estimated with

(6) $$\sigma_{UTS,predicted}=\sqrt{\frac{W_{c}}{W_{unit\_load}}}$$

in which *W_c_* is the critical SED value (Equation (1)) and $W_{unit\_load}$ is the SED value determined by means of FE analyses applying a unit load. The results thus obtained are reported in Table 3.

As can be seen, the discrepancy between the experimental and predicted tensile strength is very low.

### 4.3. TCD Critical Parameters Evaluation and Tensile Strength Prediction According to the Line Method (LM)

**Figure 6:**
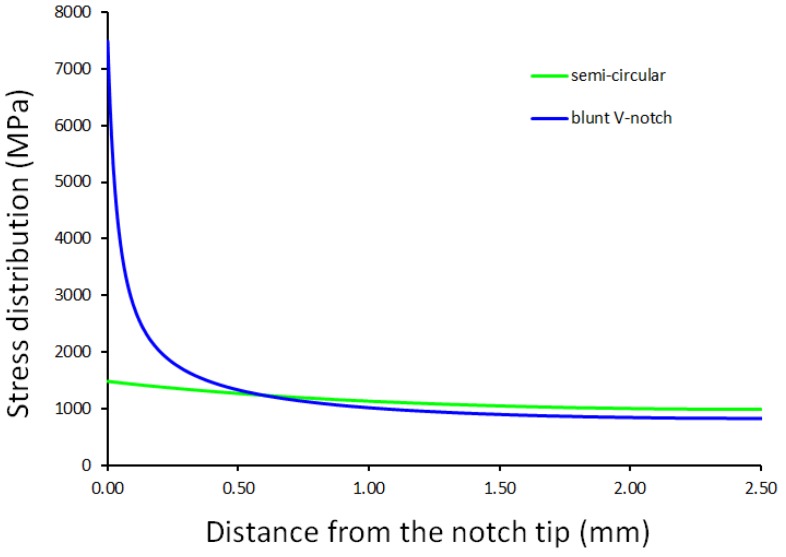
Semi-circular and blunt V-notched stress distribution ahead of the notch tip used to determine the critical TCD parameters leveraging on the point method.

The applicability of the line method requires the determination of the critical distance *L* and the inherent strength *σ*_0_. From now on, these parameters will be labeled as TCD critical parameters to differentiate them from those of SED. Being Ti-6Al-4V ductile, *σ*_0_ does not correspond to the ultimate tensile strength, and thus the TCD critical parameters need to be calibrated experimentally. This may be performed by applying the point method to two different notched geometries, as extensively described in Section 3.2. Stress fields ahead of the notch for both semi-circular and blunt V-notched specimens have been assessed using Ansys^®^ code (Figure 6). FE analyses have leveraged on the same models described in the previous section, with the only difference being that the elements size at the notch tip was about 10^−4^ mm to better describe the stress state.

At a distance from the notch tip r_c,PM_ equal to 0.6 mm, the two stress fields intersect at a stress value of 1235 MPa. As mentioned before, the point method states the critical distance *L* to be double the distance *r_c,PM_*, i.e., 1.2 mm, whilst the inherent material strength to be the stress value at which the stress distributions meet, i.e., 1235 MPa. In this case, Ti-6Al-4V is characterized by a multiplying factor T of 1.17 (see Section 3.2 and Ref. [47] for its definition). A precise interpretation of the meaning of the T parameter is still unclear, but it is related to the amount of plasticity involved in the fracture process: the higher the plasticity, the higher the T value. When determining the TCD critical parameters, tensile strength of both semi-circular and blunt V-notched specimens can be predicted by means of line method. This approach states the failure to occur for an applied load satisfying Equation (4) and the failure load has thus been predicted as the load to be applied in FE analyses in order to satisfy it. The results are gathered in Table 3, in which a comparison between the predicted values and the experimental results are also reported.

## 5. Discussions

Although both the SED approach and the LM method have shown great capability at predicting the tensile strength of different notched Ti-6Al-4V samples, providing low deviations between the experiments and the predicted results, the former seems to better estimate the failure of notched with larger radius, whereas the latter for smaller. Further experimental investigations are required to ascertain it, but the statement is in agreement with the results found by Fuentes et al. [48]. In fact, estimating the fracture loads of AL7075-T651 compact tension (CT) specimens with different notch radii, they used both SED and TCD methods, stating the SED approach to provide accurate results for large notch radii, while for small notch radii the degree of approximation of the model is noticeably conservative. On average, however, predictions provided by SED approach seem to be better. It is generally acknowledged by proponents of critical distance methods that it would be most appropriate to find the average stress over a volume in the vicinity of the hot-spot; thus, the point and line methods are seen as simplifications of a volume-based approach, i.e., SED [33]. In fact, it is generally acknowledged by proponents of critical distance methods that it would be most appropriate to find the average stress over some volume in the vicinity of the hot-spot; thus, the point and line methods are seen as simplifications of a volume-based approach, i.e., SED [33]. Moreover, SED approach is more easily applicable than TCD methods. First, the SED critical parameters have a physical meaning, being all related to material properties, whereas a precise interpretation of the inherent strength *σ*_0_ in TCD is still missing, except for brittle materials in which it corresponds to the ultimate tensile strength. Then, the SED mesh-insensitivity eases its applicability in real structures in which the need of determining accurately the stress distribution ahead of the notches to correctly perform the stress field analyses limits the TCD methods because of time-consuming FE analyses and high-performances hardware required.

Furthermore, the reliability of the SED method is still valid when material parameters from literature, i.e., fracture toughness, are used. In fact, using the more accurate critical radius obtained according to the procedure described in Section 4.2 and deeply described in Ref. [7], i.e., 1.15 mm, the results negligibly differ from those reported in Table 3. In Table 4, the predictions obtained according to the two procedures used to evaluate the critical radius are gathered together with the experimental data; in brackets, the deviations with the experiments are reported.

## 6. Conclusions

The tensile strength of semi-circular and blunt V-notched Ti-6Al-4V components have been predicted using two of the most affirmed criteria available in literature, i.e., the strain energy density approach and the line method. Both the methods provide predictions that are in good agreement with the experimental results, but the SED approach provides predictions with lower discrepancies. The discrepancies on the tensile strength using the SED are in fact lower than 1.3%, whereas with the TCD method the error for the semi-circular notched specimens is far higher, i.e., almost 8.5%. This is due to the fact that the point and line methods are seen as simplifications of a volume-based approach, as stated by the proposers of the TCD method. In addition, the SED criterion turns out to be fairly insensitive to the method used for the determination of the critical radius, leading to reliable predictions employing both published mechanical properties and FE procedures for its determination. In conclusion, both methods could be used for tensile strength assessment of notched components, regardless of the notch geometry, but the SED criterion results in better predictions and is more easily applicable because of its mesh-insensitivity and due to the faster determination of its critical parameters compared to those for the line method. 

## Figures and Tables

**Table 1:** Ti-6Al-4V tensile parameters.

*E* (MPa)	*ν*	*σ*_0.2_ (MPa)	*σ_UTS_* (MPa)
**113,000** (678)	**0.342** (0.016)	**1035** (7.5)	**1058** (4.3)

**Table 2:** Tensile strength for each of the specimen geometries, in which bold numbers represent the average values, and numbers in brackets represent the relative standard deviations.

Specimen Geometry	Tensile Strength (MPa)
Semi-circular	**1132.89** (4.82)
Blunt V-notch	**1053.08** (10.79)

**Table 3:** Prediction of tensile failure of specimens weakened by a semi-circular and blunt V-notch using the SED approach and the line method.

Specimen Geometry	Experimental Data (MPa)	SED Prediction (MPa)	SED Deviation	Line Method Prediction (MPa)	LM Deviation
Semi-circular notch	1132.89	1142.37	+0.84%	1227.37	+8.42%
Blunt V-notch	1053.08	1065.97	+1.27%	1056.56	+0.33%

**Table 4:** Comparison in the SED tensile failure assessment using the critical radius obtained according to the procedure described in Ref. [7] (“SED prediction, *R_c_* = 1.15”) or leveraging on the fracture toughness value from literature (“SED prediction, *R_c_* = 1.19”).

Specimen Geometry	Experimental Data (MPa)	SED Prediction, *R_c_* = 1.19 (MPa)	SED Prediction, *R_c_* = 1.15 (MPa)
Semi-circular notch	1132.89	1142.37 (+0.84%)	1141.84 (+0.79%)
Blunt V-notch	1053.08	1065.97 (+1.27%)	1053.08 (+1.22%)

## References

[1] Leyens C., Peters M. (2003). Titanium and Titanium Alloys: Fundamentals and Applications.

[2] Lütjering G., Williams J.C. (2007). Titanium.

[3] Elias C.N., Lima J.H.C., Valiev R., Meyers M.A. (2008). Biomedical applications of titanium and its alloys. JOM.

[4] Berto F., Lazzarin P. (2014). Recent developments in brittle and quasi-brittle failure assessment of engineering materials by means of local approaches. Mater. Sci. Eng. R.

[5] Berto F., Lazzarin P. (2009). The volume-based Strain Energy Density approach applied to static and fatigue strength assessments of notched and welded structures. Procedia Eng..

[6] Maragoni L., Carraro P.A., Peron M., Quaresimin M. (2017). Fatigue behaviour of glass/epoxy laminates in the presence of voids. Int. J. Fatigue.

[7] Peron M., Razavi S., Torgersen J., Berto F. (2017). Fracture Assessment of PEEK under Static Loading by Means of the Local Strain Energy Density. Materials.

[8] Carpinteri A. (1987). Stress-singularity and generalized fracture toughness at the vertex of re-entrant corners. Eng. Fract. Mech..

[9] Seweryn A., Łukaszewicz A. (2002). Verification of brittle fracture criteria for elements with V-shaped notches. Eng. Fract. Mech..

[10] Seweryn A., Poskrobko S., Mróz Z. (1997). Brittle Fracture in Plane Elements with Sharp Notches under Mixed-Mode Loading. J. Eng. Mech..

[11] Knesl Z. (1991). A criterion of V-notch stability. Int. J. Fract..

[12] Seweryn A. (1994). Brittle fracture criterion for structures with sharp notches. Eng. Fract. Mech..

[13] Dunn M.L., Suwito W., Cunningham S., May C.W. (1997). Fracture initiation at sharp notches under mode I, mode II, and mild mixed mode loading. Int. J. Fract..

[14] Dunn M.L., Suwito W., Cunningham S. (1997). Fracture initiation at sharp notches: Correlation using critical stress intensities. Int. J. Solids Struct..

[15] Lazzarin P., Filippi S. (2006). A generalized stress intensity factor to be applied to rounded V-shaped notches. Int. J. Solids Struct..

[16] Williams M.L. (1952). Stress singularities resulting from various boundary conditions in angular corners on plates in extension. J. Appl. Mech..

[17] Lazzarin P., Zambardi R. (2001). A finite-volume-energy based approach to predict the static and fatigue behavior of components with sharp V-shaped notches. Int. J. Fract..

[18] Gillemot F., Czoboly E., Havas I. (1985). Fracture mechanics applications of absorbed specific fracture energy: Notch and unnotched specimens. Theor. Appl. Fract. Mech..

[19] Sih G.C., Ho J.W. (1991). Sharp notch fracture strength characterized by critical energy density. Theor. Appl. Fract. Mech..

[20] Sih G.C. (1974). Strain-energy-density factor applied to mixed mode crack problems. Int. J. Fract..

[21] Sih G.C. (1973). Some basic problems in fracture mechanics and new concepts. Eng. Fract. Mech..

[22] Razavi S.M.J., Peron M., Torgersen J., Berto F., Mutignani F. (2017). Effect of hot dip galvanization on the fatigue strength of steel bolted connections. Fract. Int. Struct..

[23] Peron M., Razavi S.M.J., Berto F., Torgersen J., Marsavina L. (2017). Local strain energy density for the fracture assessment of polyurethane specimens weakened by notches of different shape. Fract. Int. Struct..

[24] Campagnolo A., Razavi S.M.J., Peron M., Torgersen J., Berto F. (2017). Mode II brittle fracture: Recent developments. Fract. Int. Struct..

[25] Razavi S.M.J., Peron M., Torgersen J., Berto F. (2017). Static Multiaxial Fracture Behavior of Graphite Components: A Review of Recent Results. Key Eng. Mater..

[26] Razavi S.M.J., Peron M., Torgersen J., Berto F., Welo T. (2017). 40CrMoV13.9 notched specimens under multiaxial fatigue: An overview of recent results. Fract. Int. Struct..

[27] Peron M., Razavi S.M.J., Berto F., Torgersen J., Mutignani F. (2017). Local strain energy density for the fatigue assessment of hot dip galvanized welded joints: Some recent outcomes. Fract. Int. Struct..

[28] Peron M., Razavi S.M.J., Berto F., Torgersen J., Colussi M. (2017). Fracture assessment of magnetostrictive materials. Fract. Int. Struct..

[29] Gallo P., Razavi S.M.J., Peron M., Torgersen J., Berto F. (2017). Creep behavior of V-notched components. Fract. Int. Struct..

[30] Ferro P., Peron M., Razavi S.M.J., Berto F., Torgersen J. (2017). The fatigue behavior of V-notches in presence of residual stresses: Recent developments and future outcomes. Fract. Int. Struct..

[31] Berto F., Campagnolo A., Lazzarin P. (2015). Fatigue strength of severely notched specimens made of Ti-6Al-4V under multiaxial loading. Fatigue Fract. Eng. Mater. Struct..

[32] Piccotin A., Marsavina L., Berto F., Negru R. (2016). Fracture parameters determination of polyurethane materials for application of SED criteria to notched components. Procedia Struct. Integr..

[33] Taylor D. (1999). Geometrical effects in fatigue: A unifying theoretical model. Int. J. Fatigue.

[34] Taylor D. (2008). The theory of critical distances. Eng. Fract. Mech..

[35] Neuber H. (1958). Theory of Notch Stresses: Principles for Exact Calculation of Strength with Reference to Structural Form and Material.

[36] Peterson R., Sines G., Waisman J.L. (1959). Notch-sensitivity. Metal Fatigue.

[37] Taylor D., Merlo M., Pegley R., Cavatorta M.P. (2004). The effect of stress concentrations on the fracture strength of polymethylmethacrylate. Mater. Sci. Eng. A.

[38] Taylor D. (2004). Predicting the fracture strength of ceramic materials using the theory of critical distances. Eng. Fract. Mech..

[39] Taylor D. (2008). The theory of critical distances: A review of its applications in fatigue. Eng. Fract. Mech..

[40] Berto F., Lazzarin P. (2009). A review of the volume-based strain energy density approach applied to V-notches and welded structures. Theor. Appl. Fract. Mech..

[41] Berto F., Campagnolo A., Gallo P. (2015). Brittle Failure of Graphite Weakened by V-Notches: A Review of Some Recent Results Under Different Loading Modes. Strength Mater..

[42] Beltrami E. (1885). Sulle condizioni di resistenza dei corpi elastici. Il Nuovo Cimento.

[43] Taylor D. (2007). The Theory of Critical Distances: A New Perspective in Fracture Mechanics.

[44] Taylor D. (2005). The Theory of Critical Distances Applied to the Prediction of Brittle Fracture in Metallic. Materials.

[45] Nasa Technical Report Center (2013). Strength, Fatigue, and Fracture Toughness of Ti-6Al-4V Liner from a Composite Over-Wrapped Pressure Vessel.

[46] Lazzarin P., Berto F., Zappalorto M. (2010). Rapid calculations of notch stress intensity factors based on averaged strain energy density from coarse meshes: Theoretical bases and applications. Int. J. Fatigue.

[47] Taylor D., Kasiri S., Brazel E. (2009). The theory of critical distances applied to problems in fracture and fatigue of bone. Fract. Int. Struct..

[48] Fuentes J., Cicero S., Berto F., Torabi A.R., Madrazo V., Azizi P. (2018). Estimation of Fracture Loads in AL7075-T651 Notched Specimens Using the Equivalent Material Concept Combined with the Strain Energy Density Criterion and with the Theory of Critical Distances. Metals.

